# Ending diagnostic odyssey by reanalysis of whole exome sequencing data: reclassification of suspected Fanconi anemia cases to dyskeratosis congenita and Diamond-Blackfan anemia

**DOI:** 10.1186/s13023-025-03928-5

**Published:** 2025-10-14

**Authors:** Eudald Tejero, María José Ramírez de Haro, Roser Pujol, Massimo Bogliolo, Benjamín Rodríguez-Santiago, Jordi Surrallés

**Affiliations:** 1 Institut de Recerca Sant Pau (IR, SANT PAU) and Joint Research Unit On Genomic Medicine, Universitat Autònoma de Barcelona (UAB)-IR SANT PAU, Barcelona, Spain; 2 Centro de Investigación Biomédica en Red de Enfermedades Raras, Instituto de Salud Carlos III (CIBERER, ISCIII), Madrid, Spain; 3 Serra Hunter Fellow, Department of Genetics and Microbiology, Faculty of Biosciences, Universitat Autònoma de Barcelona, Barcelona, Spain; 4 Hospital de La Santa Creu I Sant Pau, Barcelona, Spain

**Keywords:** WES Reanalysis, Rare Genetic Diseases, Fanconi Anemia, Diamond-Blackfan Anemia, Dyskeratosis Congenita, RPL5, TERT

## Abstract

**Background:**

Initial Whole Exome Sequencing frequently fails to resolve rare disease cases. Bioinformatic reanalysis of existing genomic data utilizes advancing knowledge to enhance diagnosis without additional testing. This study investigated six patients with clinical features consistent with Fanconi Anemia but negative chromosomal breakage tests, whose initial genetic analyses were inconclusive.

**Results:**

Whole Exome Sequencing data from these patients (collected 2005–2009) underwent comprehensive reanalysis, including single nucleotide variants, insertions/deletions, and copy number variants across genes beyond those typically associated with Fanconi Anemia. Telomere length was assessed via monochrome multiplex quantitative PCR. Reanalysis identified clinically significant variants in two patients (33.3% yield): one harboured a heterozygous pathogenic loss-of-function variant in the Diamond-Blackfan anemia gene *RPL5*, while the second exhibited compound heterozygous variants in the *TERT* gene, indicative of dyskeratosis congenita.

**Conclusions:**

This study underscores the clinical value of reanalyzing existing genomic data in unresolved suspected genetic disorders, even when phenotype-specific assays are negative. The 33.3% diagnostic yield aligns with gains from larger reanalysis studies (10–25%). Systematic reassessment after sufficient time (24 + months) for genomic advancements offers a cost-effective diagnostic approach for long-undiagnosed cases, highlighting the dynamic nature of genomic interpretation as gene-disease understanding evolves.

**Supplementary Information:**

The online version contains supplementary material available at 10.1186/s13023-025-03928-5.

## Introduction

Fanconi anemia (FA), dyskeratosis congenita (DC), and Diamond-Blackfan anemia (DBA) are inherited bone marrow failure (BMF) syndromes with high risks of bone marrow failure, leukemia, and solid tumors. The most common is FA, a rare hereditary condition characterized by defects in DNA repair mechanisms, leading to chromosomal instability and a diverse spectrum of clinical manifestations [1]. This disorder demonstrates complex inheritance patterns, with approximately 98% of cases inherited in an autosomal recessive manner, about 2% showing X-linked recessive inheritance (associated with the *FANCB* gene), and very rare instances of autosomal dominant transmission (related to the *FANCR* gene in < 1% of cases). FA typically presents in early childhood with a constellation of symptoms including congenital anomalies, progressive bone marrow failure, and significantly elevated risk of hematologic and solid malignancies [2].

The diagnostic approach for FA primarily relays on the chromosomal breakage test, which serves as the distinctive diagnostic marker for the condition. This test involves exposing patient cells to DNA cross-linking agents such as diepoxybutane (DEB) or mitomycin C (MMC) to evaluate chromosomal fragility [2]. While this test is the cornerstone of diagnosis, genetic testing plays a crucial complementary role in confirming the diagnosis and identifying the specific genetic variant responsible [3]. It is particularly valuable in cases of somatic mosaicism, where chromosomal breakage tests using blood cells may yield normal results, necessitating additional testing on fibroblasts [4, 5]. Despite thorough testing, some individuals exhibit clinical features strongly suggestive of FA but have negative chromosome fragility tests and no identifiable variants in known FA-related genes.

Next Generation Sequencing (NGS) is a powerful tool for the diagnosis of genetic disorders. In cases where initial genetic testing, such as Whole Exome Sequencing (WES), does not yield a diagnosis, reanalysis of the generated NGS data has emerged as a powerful strategy to increase the diagnostic yield without requiring additional sequencing [6, 7]. This approach holds particular relevance as genomic interpretation represents a dynamic process that evolves with the continuous discovery of new disease-causing genes and the development of improved bioinformatics tools and analytical methodologies. Multiple studies have demonstrated that routine reanalysis over time significantly improves diagnostic rates, with a recent systematic review and meta-analysis indicating an overall diagnostic yield of 10% in previously undiagnosed cases [8]. More specifically, research on dystonia patients has shown that genome sequencing data reanalysis increased the diagnostic yield from 11.7% to 18.9%, with potential further extension up to 22.5% [9].

Several interconnected factors contribute to the increased diagnostic success achieved through reanalysis. Literature updates and newly established gene-disease associations represent primary drivers of new diagnoses, as the rapid pace of genetic discovery means that revisiting existing data allows for the identification of genes newly linked to disease after the initial analysis [10]. The constant expansion of genetic knowledge creates opportunities for reinterpretation that were simply unavailable at the time of initial testing. Concurrently, improvements in bioinformatics tools and analytical methods substantially enhance the ability to detect and interpret genetic variants [10]. Updated annotation databases and more sophisticated variant calling algorithms can reveal previously missed or misclassified variants, as exemplified by cases where pathogenic (P) variants were initially overlooked due to annotation to less relevant isoforms [10].

The accumulation of knowledge regarding genotype–phenotype correlations and the potential for phenotype expansion over time can significantly aid in the interpretation of molecular findings during reanalysis [11]. Detailed re-evaluation of the patient’s clinical information becomes crucial in this context, as evolving understanding of genetic disorders may reveal that a patient’s symptoms align with recently described manifestations of particular genetic condition [12]. Furthermore, collaborative research efforts and data sharing platforms facilitate the validation of candidate variants and the identification of novel gene-disease associations. International collaborations, such as those demonstrated in the European Solve-RD study, have yielded new diagnoses through combined expertise that would not have been possible through isolated analysis [12].

Given these substantial advantages, recent literature increasingly recommends the routine reanalysis of unsolved NGS data after a period of time [7]. Studies suggest that a timeframe of 24 months or more after the initial analysis may enhance effectiveness by allowing sufficient time for new discoveries and advancements [13]. Implementation of reanalysis programs also faces practical challenges, including resource allocation considerations, data storage requirements, and ethical questions regarding consent and communication of new findings [6]. Nevertheless, the potential benefits are substantial, particularly for patients with rare or complex presentations.

In the present study, a reanalysis of WES data was performed on six patients who presented clinically with a suspected FA phenotype. The investigation aimed to identify genetic variants that could explain their conditions by examining genes beyond those traditionally associated with FA. This approach exemplifies the utility of reanalyzing genomic data in cases where initial genetic testing did not yield a diagnosis, potentially providing answers for patients who have long sought explanations for their medical conditions and opening avenues for more targeted clinical management strategies.

## Materials and methods

### Patients

The study included six patients, including one male (16.7%) and five females (83.4%), who were referred for genetic testing based on initial clinical suspicion of FA. The relevant clinical history for each patient is summarised as follows: Patient 1: Diagnosed with immune thrombocytopenic purpura (ITP) at age 10 (2001). Subsequently developed bilateral interstitial pneumonia with respiratory failure and recurrent aphthous ulcers of the lips and oral mucosa. Chronic diarrhoea led to a diagnosis of irritable bowel syndrome. In late 2003, hematological evaluation revealed total bone marrow aplasia. Patient 2: Diagnosed in 2010 with mild leukopenia-neutropenia anemia and a mean corpuscular volume (MCV) within the upper normal range for age. Patient 3: A sample from this patient was referred for FA exclusion in 2007. Due to a lack of available clinical data from the time of the fragility study and the inability to contact the physician, further clinical details are unavailable. Patient 4: Moderate thrombocytopenia was detected at age two. Additional clinical features included microcephaly, café-au-lait spots, epileptic episodes, and developmental delay. The mother presented with microcephaly, and there was a family history of epilepsy. The father was reportedly healthy. The patient’s thrombocytopenia worsened, necessitating a transplant that was refused by the family. The patient died shortly thereafter. Patients 5 and 6 (Siblings): These siblings presented with macrocytic anemia, short stature, and a syndromic appearance. Their father had a childhood diagnosis of FA. Patient 6 was diagnosed with FA in 1988, while Patient 5’s diagnosis remained uncertain. Patient 6 exhibited a more severe phenotype, including agenesis of the left kidney, bilateral cryptorchidism, hypertelorism, microcephaly, winged auricles, and psychomotor retardation. Additional clinical data for all patients is provided in Table 1.

**Table 1:** Demographic and chromosome fragility data and clinical features of suspected Fanconi anemia studied patients

Patient	Birth date	Sex	Ethnicity	Year of first diagnosis	Fragility study date	% Aberrant cells (DEB)	First clinical phenotype	Disease course	Family history
1	06/08/1991	F	Caucasian	2001	25/01/2006	0	Immune thrombocytopenic purpura	Bone marrow aplasia in 2003 (allogeneic transplant from a family donor), bilateral interstitial pneumonias with associated respiratory failure (lung transplant in 2021), aphthosis, irritable bowel syndrome, severe neutropenia in the most recent clinical report	-
2	04/04/2001	F	Caucasian	2010	01/03/2006	2	Mild leukopenia-neutropenia and anemia	Anemia was normalized, leukopenia maintained, medical discharge in 2015	-
3	26/12/2006	F	Caucasian	-	31/07/2007	4	Referred for "FA exclusion in 2007"	Not available	-
4	10/02/2001	F	Gypsy	2003	06/11/2007	9	Moderate thrombocytopenia, cafe-au-lait spots, microcephaly, epileptic episodes and mild mental retardation	Severe megakaryocyte thrombocytopenia in 2014 (transplant required, but the family refused, and the patient died shortly afterward)	Healthy father, mother with microcephaly and family history of epilepsy
5*	04/12/1979	F	Caucasian	1988	29/01/2009	11	Macrocytic anemia, short stature and a syndromic appearance	-	Paternal family history: FA diagnosed in childhood and subsequently ruled out
6*	21/06/1981	M	Caucasian	1988	29/01/2009	13	Macrocytic anemia, short stature and syndromic appearance, agenesis of the left kidney, bilateral cryptorchidism, hypertelorism, microcephaly, winged auricles, and psychomotor retardation	–	Paternal family history: FA diagnosed in childhood and subsequently ruled out

### Standard exome sequencing and analysis

Genomic DNA samples were isolated and obtained from whole blood following standard procedures. WES for four of these individuals (designated as Patients 1 to 4) was carried out at *Sistema Genómicos* (Valencia, Spain) during July 2013, following their standard protocols. Subsequently, in early 2014, samples from the remaining two patients (Patients 5 and 6) were sent to the GATC Biotech sequencing center (Konstanz, Germany).

### Sequencing data reanalysis

**Fig. 1:**
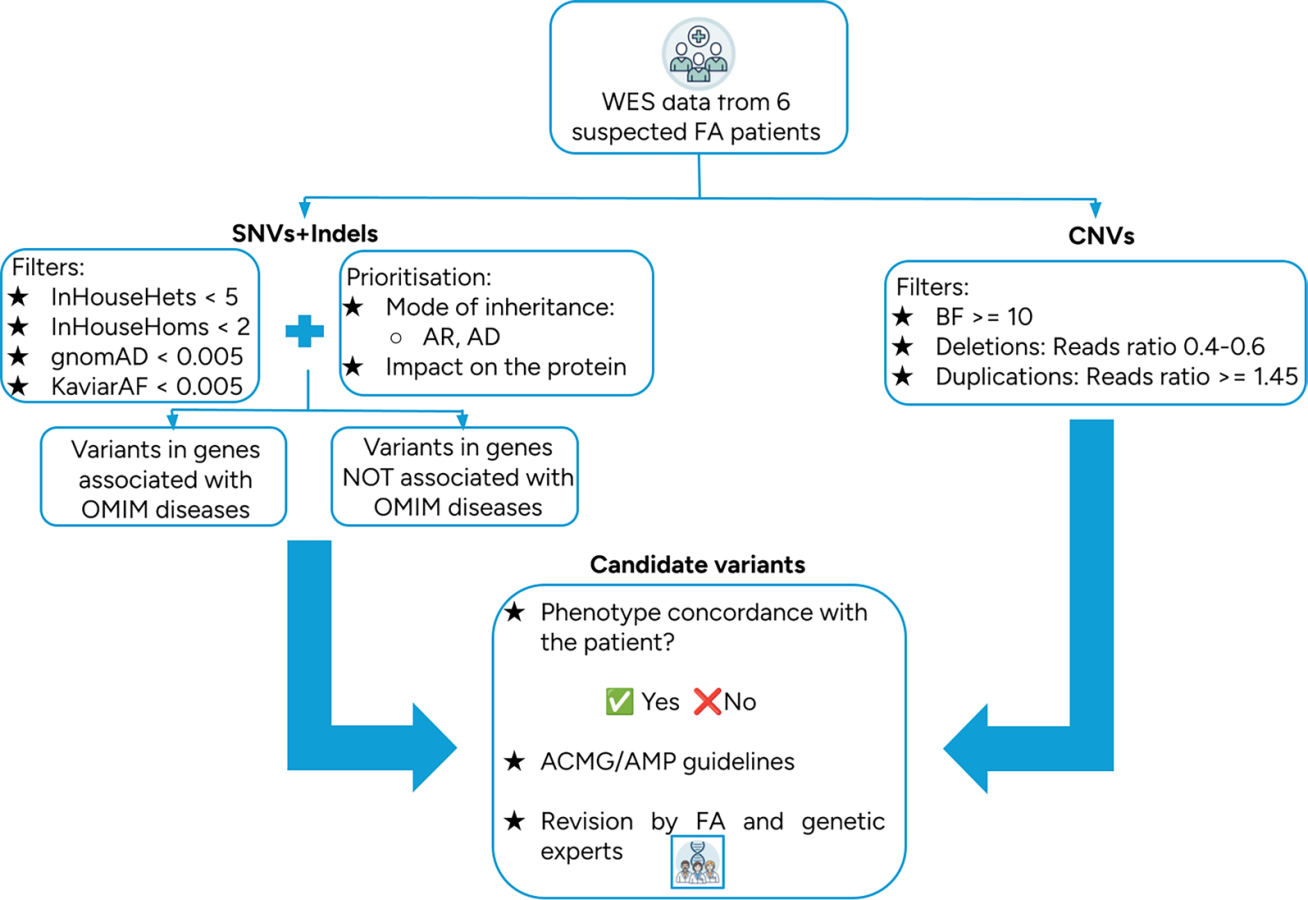
Workflow of the reanalysis. Six patients with suspected FA clinical phenotype but with negative chromosome fragility test and no FA-related variants identified after WES were selected for reanalysis. SNVs and indels were filtered based on < 0.5% allele frequency in variant databases as well as below 2 homozygotes or 5 heterozygotes individuals at our in-house database and prioritized by the disease mode of inheritance and effect on the encoded protein. CNVs were analyzed with ExomeDepth and filtered by BF and reads ratio. Finally, candidate variants were obtained and discussed in a committee with FA and genetic experts to elucidate their implication in their respective patients’ condition. Variants were classified according to ACMG/AMP guidelines

The six patients had inconclusive results from the initial analysis focused on known FA genes and related ones. Sequencing fastq files were recovered and underwent a systematic reanalysis as it is shown in Fig. 1. Briefly, sequence reads were aligned against the GRCh37 human reference genome assembly using the Burrows-Wheeler Aligner (BWA) [14] and Picard tool from Broad Institute (http://broadinstitute.github.io/picard/). Genome Analysis Toolkit (GATK) [15] was employed for the calling of single nucleotide variants (SNVs) and indels while functional annotation was performed with the use of ANNOVAR [16]. Copy number variants (CNVs) were analyzed using RStudio and following packages: ExomeDepth [17], Genomic Ranges and IRanges [18]. Sequencing quality was assessed using FastQC (https://www.bioinformatics.babraham.ac.uk/projects/fastqc/). Quality coverage metrics were obtained with Picard: the mean exome coverage was 86.1X, the median coverage was 70.3X and the percentage of accumulated coverage at least at 20X was 87%. A virtual panel of FA-related genes (**Table S1**) was applied to filter identified variants in the first genetic analysis. Two main groups of variants were considered: 1) variants in genes associated with OMIM diseases; 2) variants in genes not associated with OMIM diseases. Variants of the first group were analysed and in case no candidate variants were found, the second group was examined considering available literature to elucidate if a gene could explain the clinical phenotype of the patient. As the siblings (patients 5 and 6) had similar clinical features, variants shared in both patients were also deeply assessed. Variant prioritization to identify potential candidate disease variants was carried out by using different resources, including allele frequency from public databases gnomAD [19] and Kaviar [20] and our in-house database of individuals from the same geographical area; impact on protein (missense, in-frame, splicing, frameshift, nonsense, CNV) and inheritance model. For missense rare variants, in silico prediction REVEL program was used to assess potential impact on protein function. In the case of CNVs, variants with a Bayes factor (BF) below 10 were discarded. Only deletions with a reads ratio between 0.4 and 0.6 and duplications with a reads ratio equal or higher than 1.45 were considered. Reciprocal overlapping intervals between CNVs of the six patients and the siblings were analyzed employing BEDTools [21]. Final candidate variants were reviewed by an expert team of clinicians and geneticists to elucidate their involvement in the patient’s phenotype. The ACMG/AMP guidelines were considered to give a final classification [22]. Candidate variants were confirmed using Sanger sequencing. Topoisomerase-based cloning assay was used to identify variants in *trans* when no family relatives were available.

### Telomere length and chromosome fragility

Telomere length was measured as previously described by Cawthon (2009) [23] with some modifications. Briefly, genomic DNA was extracted from whole blood, purified using the DNeasy® PowerClean® Pro Cleanup Kit (Qiagen, 12,997–50), and quality controlled. Telomere length was measured using the monochrome multiplex qPCR on a 7900HT Applied Biosystems instrument. Reactions included telomere and albumin primers and PowerUp™ SYBR™ Green Master Mix (Applied Biosystems). Each sample was analyzed in triplicate wells on two separate plates. A five-point standard curve of control DNA was used to calculate the T/S ratio. Data analysis was performed using the Design and Analysis software (v.2.6.0; Thermo Fisher)**.** Chromosome fragility was assessed in all patients using DEB induction, as previously described [24].

## Results

**Fig. 2:**
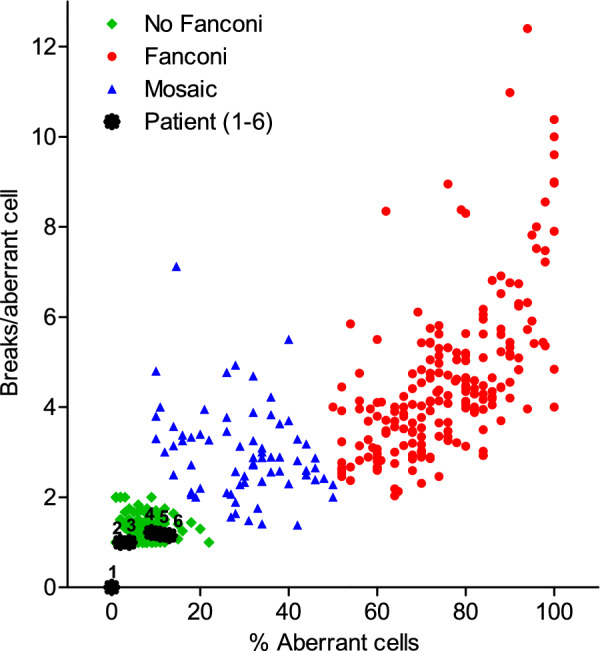
Chromosome fragility results. The plot shows a graphical representation of the relative position of cases (suspected FA patients 1–6, with numbers over asterisk symbols in the plot) when considering the distribution of historical data from our laboratory in terms of percentage of aberrant cells and mean number of breaks per cell (all induced by DEB at 0,1 μg/ml). Green signals correspond to non-Fanconi patients, red are FA patients, blue represent mosaic FA patients, asterisk symbols correspond to analysed patients in this work. Mosaic patients are those FA patients with a percentage of aberrant cells below 50%

Chromosome fragility and G2 arrest data (G2 data not shown) were incompatible with a classical FA phenotype (Fig. 2), disregarding FA as initial diagnosis. Patients (1–6) showed values overlapping with those from non-Fanconi patients’ group (Fig. 2).

**Fig. 3:**
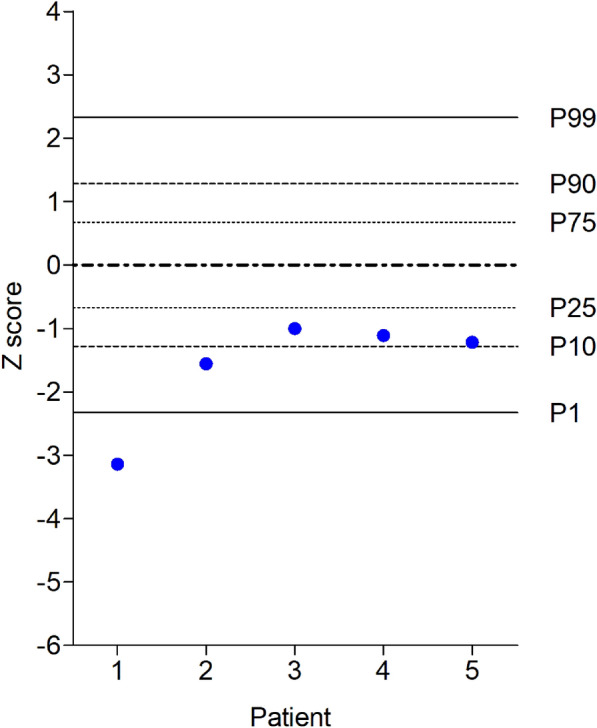
Telomere length in suspected Fanconi anemia patients. Patients 1–5 with suspected FA represented in x-axe. Patient 6 not analyzed due to technical issues. Horizontal lines represent the mean (0), and the values -2.326, -1.282, -0.674, + 0.674, + 1.282, and + 2.326 correspond to the standard deviations from the mean, equivalent to the 1st, 10th, 25th, 75th, 90th, and 99th percentiles (P1-P99) for control individuals

Consistently with negative chromosome fragility test, no variants were identified in none of the known FA associated genes (**Table S1**) in the standard analysis. The performed reanalysis of sequencing data in other genes identified three clinically significant variants in two out of six patients (33.4%) (Table 2). Patient 1 showed two variants in the *TERT* gene: The first one was a nonsense variant creating a premature stop codon and a likely loss of function of the protein codified by the allele (NM_198253.3, c.337G > T:p.Glu113*). The second variant consisted in a missense change (c.193C > A:p.Pro65Thr). The variants were confirmed by Sanger sequencing and the topoisomerase-based cloning analysis showed that they were in *trans*. Given that *TERT* mutations are associated to telomere shortening, telomere length was measured in Patient 1 and the results were compatible with a telomeropathy (Fig. 3), exhibiting the most pronounced telomere shortening, with a z score considerably lower than the control mean, below percentile 1.

**Table 2:** Clinically relevant variants identified after reanalyzing exome sequencing data

Patient	Sex of the patient	Gene	Reference transcript	Variant	Aminoacid change	Variant type	Variant classification	ACMG evidences
1	Female	*TERT*	NM_198253.3	c.337G > T	p.Glu113*	Nonsense	LP	PVS1, PM2
				c.193C > A	p.Pro65Thr	Missense	LP	PM2, PP2, PM3
2	Female	*RPL5*	NM_000969.5	c.175_176del	p.Asp59Tyrfs*5 3	Frameshift	P	PVS1, PM2, PS4, PP5

In Patient 2, a *RPL5* frameshift variant (NM_000969.5: c.175_176del; p.Asp59Tyrfs*53) was identified. This *RPL5* variant, which introduces a premature stop signal might cause loss of function, either by a shortened, non-functional protein or by triggering nonsense-mediated decay (NMD) of the mRNA. Telomere length analysis in Patient 2 revealed shortened telomeres with a z score below percentile 10 but above percentile 1 (Fig. 3); thus, telomeropathy in this patient based on this parameter was discarded.

In the remaining four patients, no clinically significant variants were identified in genes with known disease associations and concordant with their observed phenotype. Although patients 3 to 5 have short telomeres (below percentile 25) (Fig. 3), they exhibit a z score above the 1st percentile, suggesting incompatibility with a telomeropathy. Additionally, the analysis of non-OMIM genes failed to reveal a novel candidate gene that could be associated with the phenotype, according to existing evidence and knowledge. Rare variants of uncertain significance (VUS) with an extreme low population frequency, predicted to be highly deleterious and/or identified in both siblings, and residing in genes associated to clinical phenotypes or functions partially overlapping patients’ features were identified (**Table S2**), but without enough evidence to classify them as P or likely pathogenic (LP) following ACMG/AMP guidelines.

## Discussion

Reanalysis of WES data led to the identification of disease-causing variants in patients 1 and 2. In Patient 1 a nonsense variant (NM_198253.3: c.337G > T; p.Glu113*) and a missense change (NM_198253.3: c.193C > A; p.Pro65Thr) were identified in the *TERT* gene. The nonsense variant shows an extremely low frequency in gnomAD and loss of function is a known mechanism of disease in the gene [25]. According to this evidence, the variant was classified as LP. The missense change also presents an extremely low frequency in gnomAD and the gene shows a low rate of benign missense mutations which are a common mechanism of disease. Although the variant was found in a patient with aplastic anemia also carrying a second missense change [26], it was classified as VUS as there was not enough available evidence to confirm its pathogenicity. *TERT* gene is associated with autosomal dominant or recessive DC, a telomere disease. While there is significant overlap in the clinical features of DC regardless of inheritance pattern, some nuances can exist: severe forms of DC, like Hoyeraal-Hreidarsson syndrome, which often involves cerebellar hypoplasia and developmental delay, can be associated with both autosomal recessive and dominant inheritance, depending on the specific gene involved. The age of onset and the rate of progression can vary between individuals and families, and this variation is not strictly tied to the inheritance pattern but more to the specific gene and mutation. The classic triad of nail dystrophy, lacy reticular pigmentation, and oral leukoplakia can be present in both autosomal recessive and dominant forms, but it is important to note that not all individuals with DC present with all three features. Both autosomal recessive and autosomal dominant forms of DC are characterized by shortened telomeres. The degree of telomere shortening can vary and may correlate somewhat with disease severity, but this isn’t exclusively determined by the inheritance pattern. There is significant phenotypic variability in DC, even within families with the same mutation. Anticipation can occur in DC, particularly in autosomal dominant forms, where successive generations may exhibit earlier onset and more severe symptoms. The studied patient presents severe telomere shortening (Fig. 3) and developed a bone marrow aplasia which required an allogeneic transplant in 2003. In 2021 she was diagnosed with interstitial lung disease and received a lung transplant. The patient has also presented chronic diarrhea that was related to irritable bowel syndrome. In the most recent clinical report, she manifested severe neutropenia. The variants were confirmed by Sanger sequencing and the topoisomerase-based cloning analysis showed that they were in *trans*. This evidence gives the variant the ACMG/AMP PM3 evidence, that is a variant occurring in *trans* with a P variant for a recessive disorder, therefore it can be classified as LP. The DC caused by *TERT* gene has been associated to dominant and recessive inheritance patterns, with the latter having additional characteristics such as leukoplakia, failure to thrive, cerebellar hypoplasia, microcephaly and developmental delay [27] not presented in our patient. The telomere length of this patient was 2,608 base pairs (bp) at 14.3 years of age, with a z score of -3.141, significantly below the 1st percentile (-2.326). This indicates severe telomere shortening, consistent with a telomere disease (Fig. 3).

In the case of the *RPL5* variant found in Patient 2 (NM_000969.5: c.175_176del; p.Asp59Tyrfs*53), it was classified as P based on the following evidence: loss-of-function variant in *RPL5,* a known mechanism in patients [28, 29], the deletion is extremely rare (no frequency at gnomAD) but it has been observed in individuals with DBA [28, 30, 31]. DBA is an inherited red blood cell aplasia that usually presents in the first year of life. The main features are normochromic macrocytic anemia, reticulocytopenia, and nearly absent erythroid progenitors in the bone marrow. Patients show growth retardation, and approximately 30 to 50% have craniofacial, upper limb, heart, and urinary system congenital malformations. Most patients have increased mean corpuscular volume, elevated erythrocyte adenosine deaminase activity, and persistence of hemoglobin F. However, some DBA patients do not exhibit these findings, and even in the same family, symptoms can vary between affected family members. The studied patient showed a mild disease phenotype as in 2010 she had anemia with a MCV at the normal-high limit for her age and a mild leukopenia-neutropenia. In the most recent clinical report, the anemia was normalized while she maintained the mild leukopenia. This can be explained by the clinical heterogeneity of the disease [32]: individuals with the same variant identified in our study exhibit different clinical characteristics [28]. In some cases, affected individuals within the same family may show mild anemia or even no evidence of clinical disease [28, 33]. In addition, this patient had shortened telomeres compared with controls (at 10.9 years of age was 7,116 bp, which is below the 10th percentile (-1.282) but above the 1st percentile (-2.326), with a z score of -1.557, although this was not as severe as in patients with DC. This finding is consistent with the diagnosis of DBA reported by Alter et al. (2015) [34].

Despite a comprehensive reanalysis of OMIM and non-OMIM genes, no further variants of interest were detected in the remaining patients. The absence of findings is likely multifactorial, with one key factor being the limitations of the short-read WES methodology used. Short-read sequencing is known to have reduced sensitivity for structural variants (SVs), due to the difficulty in assembling reads across complex genomic rearrangements, especially within repetitive regions [35]. Furthermore, WES’s exon-centric design hinders the detection of variants in critical non-coding regions [36], such as deep intronic and regulatory sequences. Other limitations, including uneven coverage in GC-rich regions [37] and the difficulty in detecting small CNVs [38], also contribute to the potential for missed variants. Long-read sequencing could overcome these limitations by effectively resolving complex and repetitive regions, leading to improved detection of SVs and other variant types, including SNVs [39–43]. Similarly, Whole Genome Sequencing (WGS) provides a more comprehensive genomic view, allowing for the identification of SVs and CNVs frequently missed by WES and enabling the exploration of potentially P variants in non-coding regions [44–46]. Another contributing factor may be the limited knowledge of many reviewed non-OMIM genes, which resulted in numerous rare VUS. In addition, there is also a possibility of a disease caused by the combined effects of multiple genes, each contributing a small risk. WES may identify some of these risk variants, but it is difficult to determine their overall contribution to the disease.

The findings of this study highlight the clinical benefit of reanalyzing WES data in patients with undiagnosed genetic conditions, particularly after initial, targeted analyses focusing on canonical FA genes have proven negative, while phenotype-specific assays also yield non-diagnostic results. Despite the modest number of studied patients, the percentage of new diagnoses identified through a subsequent, broader analysis of WES data, which included a more comprehensive panel of genes implicated in BMF syndromes, aligns with the diagnostic yields reported in larger cohorts (10–25%). This work underscores the importance of expanding the scope of genetic investigation beyond traditional FA genes in patients presenting suspected FA features but normal chromosome fragility. We propose that future studies implement an expanded gene panel in these phenotypes during the reanalysis of WES data. This strategy is expected to enhance diagnostic rates by detecting a wider array of genetic causes, ultimately leading to improved patient care.

## Conclusions

Systematic reanalysis of WES data at appropriate intervals is a cost-effective and crucial approach for long-undiagnosed conditions. In this study we achieved a 33.3% diagnostic yield in a small cohort of six patients initially suspected of FA who had remained undiagnosed through conventional methods.

In our study, we diagnosed patients with DC and DBA, and our findings were corroborated by integrating them with clinical and functional data, including telomere length measurements.

Our work highlights the importance of allowing sufficient time for clinical databases to expand, variant annotation to improve, and novel disease-gene relationships to be identified. In this context, WES reanalysis becomes an indispensable tool for maximizing diagnostic accuracy and delivering personalized care to patients who have long sought answers. Our study also recalls the very close clinical, cellular, and molecular links between these three syndromes as previously discussed [47].

## Supplementary Information

Additional file 1.

Additional file 2.

## Data Availability

The datasets used and/or analysed during the current study are available from the corresponding author on reasonable request.
