# Supplementary material for: Ending diagnostic odyssey by reanalysis of whole exome sequencing data: reclassification of suspected Fanconi anemia cases to dyskeratosis congenita and Diamond-Blackfan anemia
Source: Orphanet J Rare Dis. 2025 Oct 14;20:511. doi: 10.1186/s13023-025-03928-5 (PMC12522949; doi:10.1186/s13023-025-03928-5)
Supplement: Supplementary file 1 — Additional file 1. [file 13023_2025_3928_MOESM1_ESM.pdf]

Supplementary Table 1. Fanconi anemia-related genes considered in the standard analysis.

Gene/Locus	Gene/Locus MIM number	Location	Fanconi anemia complementation group	Inheritance	Phenotype MIM number
MAD2L2	604094	1p36.22	V	AR	617243
UBE2T	610538	1q32.1	T	AR	616435
PHF9	608111	2p16.1	L	AR	614083
FANCD2	613984	3p25.3	D2	AR	227646
FANCE	613976	6p21.31	E	AR	600901
XRCC2	600375	7q36.1	U	AR	617247
XRCC9	602956	9p13.3	G	AR	614082
FANCC	613899	9q22.32	C	AR	227645
FANCF	613897	11p14.3	F	AR	603467
BRCA2	600185	13q13.1	D1	AR	605724
RAD51	179617	15q15.1	R	AD	617244
FANCI	611360	15q26.1	I	AR	609053
SLX4	613278	16p13.3	P	AR	613951
ERCC4	133520	16p13.12	Q	AR	615272
PALB2	610355	16p12.2	N	AR	610832
RFWD3	614151	16q23.1	W	AR	617784
FANCA	607139	16q24.3	A	AR	227650
BRCA1	113705	17q21.31	S	AR	617883
RAD51C	602774	17q22	O	AR	613390
BRIP1	605882	17q23.2	J	AR	609054
FANCB	300515	Xp22.2	B	XLR	300514
FANCM	609644	14q21.2	M	AR	618096

AR: Autosomal Recessive; AD: Autosomal Dominant; XLR: X-Linked Recessive.
