# Supplementary material for: Ending diagnostic odyssey by reanalysis of whole exome sequencing data: reclassification of suspected Fanconi anemia cases to dyskeratosis congenita and Diamond-Blackfan anemia
Source: Orphanet J Rare Dis. 2025 Oct 14;20:511. doi: 10.1186/s13023-025-03928-5 (PMC12522949; doi:10.1186/s13023-025-03928-5)
Supplement: Supplementary file 2 — Additional file 2. [file 13023_2025_3928_MOESM2_ESM.pdf]

Supplementary Table S2. Summary of variants of uncertain significance identified across the patient cohort after reanalyzing exome sequencing data.

Patient/s	Gene	RefSeqTranscript	c.DNA<br>p.change	gnomAD	REVEL	MIM Number
3	*PIEZO1*	NM_001142864.4	c.7184C>T<br>p.Ala2395Val	0.0001075	0.196	611184
3	*EPAS1*	NM_001430.5	c.2259C>A<br>p.Asp753Glu	0.018	0.039	603349
3	*THPO*	NM_000460.4	c.914C>T<br>p.Thr305Met	0.00001998	0.049	600044
3	*CFB*	NM_001710.6	c.1133A>C<br>p.Asn378Thr	0.000271	0.405	138470
3	*MUC15*	NM_024690.2	c.37019A>G<br>p.Asn12340Ser	0	0.019	171860
3	*MUC16*	NM_024690.2	c.4181C>T<br>p.Thr1394Ile	0	0.086	606154
3	*CEBPZ*	NM_005760.3	c.61del<br>p.Ala21Glnfs*2	0	-	612828
3	*CEBPZ*	NM_005760.3	c.60G>C<br>p.Glu20Asp	0	0.109	612828
3	*ZDXA*	NM_007156.5	c.403_404del<br>p.Cys135Leufs*42	0.0003536	-	300235
3	*ZDXA*	NM_007156.5	c.401_402del<br>p.Gly134Valfs*43	0	-	300235
4	*RAD50*	NM_005732.4	c.2492A>G<br>p.Glu831Gly	0.00002787	0.361	604040
5	*HRCT1*	NM_001039792	c.317_318insCA<br>p.H107Tfs*52	0	-	-
5	*ANKLE1*	NM_001278444.2	c.1772del<br>p.Leu591Cysfs*18	0	-	619348
6	*ABCA8*	NM_001288985.2	c.1181del<br>p.Gly394Alafs*6	0.00004088	-	612505
5, 6	*PFKL*	NM_001002021.3	c.106G>A<br>p.Gly36Ser	0.000004008	0.2	171860
5, 6	*GTDC1*	NM_001284238.3	c.26G>A<br>p.Trp9*	0.0009865	-	610165
5, 6	*CRY2*	NM_021117.5	c.1505G>A<br>p.Arg502Gln	0	0.506	603732
5, 6	*FCGBP*	NM_003890.2	c.753C>A<br>p.His251Gln	0	0.007	617553
5, 6	*KIF20B*	NM_001284259.2	c.2135G>C<br>p.Cys712Ser	0	0.084	605498
5, 6	*NCAM1*	NM_001242607.2	c.1118G>A<br>p.Gly373Asp	0.0000297	-	116930
5, 6	*TPM4*	NM_003290.3	c.163C>G<br>p.Arg55Gly	0	0.788	600317
